# Hypercytotoxicity and Rapid Loss of NKp44^+^ Innate Lymphoid Cells during Acute SIV Infection

**DOI:** 10.1371/journal.ppat.1004551

**Published:** 2014-12-11

**Authors:** Haiying Li, Laura E. Richert-Spuhler, Tristan I. Evans, Jacqueline Gillis, Michelle Connole, Jacob D. Estes, Brandon F. Keele, Nichole R. Klatt, R. Keith Reeves

**Affiliations:** 1 Center for Virology and Vaccine Research, Beth Israel Deaconess Medical Center, Boston, Massachusetts, United States of America; 2 Department of Pharmaceutics, Washington National Primate Research Center, University of Washington, Seattle, Washington, United States of America; 3 Division of Immunology, New England Primate Research Center, Harvard Medical School, Southborough Campus, Southborough, Massachusetts, United States of America; 4 AIDS and Cancer Virus Program, Leidos Biomedical Research, Inc., Frederick National Laboratory, Frederick, Maryland, United States of America; Emory University, United States of America

## Abstract

HIV/SIV infections break down the integrity of the gastrointestinal mucosa and lead to chronic immune activation and associated disease progression. Innate lymphoid cells (ILCs), distinguishable by high expression of NKp44 and RORγt, play key roles in mucosal defense and homeostasis, but are depleted from gastrointestinal (GI) tract large bowel during chronic SIV infection. However, less is known about the kinetics of ILC loss, or if it occurs systemically. In acute SIV infection, we found a massive, up to 8-fold, loss of NKp44^+^ILCs in all mucosae as early as day 6 post-infection, which was sustained through chronic disease. Interestingly, no loss of ILCs was observed in mucosa-draining lymph nodes. In contrast, classical NK cells were not depleted either from gut or draining lymph nodes. Both ILCs and NK cells exhibited significantly increased levels of apoptosis as measured by increased Annexin-V expression, but while classical NK cells also showed increased proliferation, ILCs did not. Interestingly, ILCs, which are normally noncytolytic, dramatically upregulated cytotoxic functions in acute and chronic infection and acquired a polyfunctional phenotype secreting IFN-γ, MIP1-β, and TNF-α, but decreased production of the prototypical cytokine, IL-17. Classical NK cells had less dramatic functional change, but upregulated perforin expression and increased cytotoxic potential. Finally, we show that numerical and functional loss of ILCs was due to increased apoptosis and ROR γt suppression induced by inflammatory cytokines in the gut milieu. Herein we demonstrate the first evidence for acute, systemic, and permanent loss of mucosal ILCs during SIV infection associated with reduction of IL-17. The massive reduction of ILCs involves apoptosis without compensatory *de novo* development/proliferation, but the full mechanism of depletion and the impact of functional change so early in infection remain unclear.

## Author Summary

HIV-1 has long been shown to deplete CD4+ T cells and disrupt barrier integrity in the gastrointestinal tract, but effects on other subpopulations of lymphocytes are less well described. A recently identified subpopulation of mucosa-restricted cells, termed innate lymphoid cells (ILCs) is thought to play critical roles in maintaining homeostasis in the gastrointestinal tract and mucosal pathogen defense. Although previous work from our laboratory and others have shown SIV infection of rhesus macaques can deplete ILCs in some parts of the gastrointestinal tract, systemic as well as kinetic effects were unclear. In this report we show that ILCs, but not classical NK cells are systemically depleted during infection and also acquire cytotoxic capabilities. Furthermore, our data is the first to indicate that this important subset of innate cells is depleted acutely, permanently, and systemically during SIV infection of rhesus macaques as a model for HIV-1 infection. Given the important role of ILCs in maintaining gut homeostasis these findings could have significant implications for the understanding and treatment of HIV-induced disease.

## Introduction

During acute infection, the gastrointestinal (GI) tract is a primary target site for HIV-1 and SIV replication [1]–[4]. CD4^+^T cells are rapidly infected and depleted and the mucosal epithelial barrier is compromised. These early events after infection generally set the pace of disease progression, and while subsequent microbial translocation and immune activation drive ongoing disease, the early events in the mucosae following infection remain incompletely understood [2], [3], [5]–[7].

A growing number of reports indicate that innate lymphoid cells (ILCs) play critical roles in maintaining mucosal epithelial integrity, tissue remodeling and repair, and defense against intestinal pathogens [8]–[12]. ILCs are a heterogeneous group of the lymphoid lineage, but depend on the helix-loop-helix transcription factor inhibitor of DNA binding 2 (Id2), the common γ-chain receptor and IL-7 for their development [13]–[17]. ILCs are divided into three groups in mice and humans, based on their expression of cell surface markers, functional characteristics and transcriptional regulation. Group 1 ILCs (ILC1) contain natural killer (NK) cells, which are cytotoxic, produce IFN-γ and depend on T-bet for their development; group 2 ILCs (ILC2) are innate IL-5- and IL-13-producing cells and depend on transcription factor GATA-3 for lineage commitment; group 3 ILCs (ILC3) produce IL-22 and/or IL-17 and depend on RORγt for development [18]–[22]. Interestingly, development of both ILC1 and ILC3 require IL-7, but additive IL-β drives differentiation to ILC3. In contrast, addition of IL-12, IL-15, or IL-18 in combination with IL-7 drives differentiation toward ILC1. Although the general features of ILCs are conserved in mice and humans, no specific uniform nomenclature for ILCs has been ascribed in rhesus macaques, due to a lack of identification of each lineage. Previously, we identified NKp44^+^ILCs from rhesus macaques and found them to be restricted to mucosal tissues, express high levels of RORγt, and produce IL-17 [23], making them most likely analogous to ILC3. Furthermore, during chronic SIV infection, others and we have shown that NKp44^+^ILCs are reduced in the GI tract and IL-17 production is suppressed [23]–[27]. However, these studies were performed primarily in limited tissues and in chronically SIV-infected animals. The systemic effects of SIV infection and kinetics of loss are unknown.

The role(s) of NK cells in HIV pathogenesis and disease remains controversial. Studies on highly exposed individuals who remain seronegative (HESN), including intravenous drug users and heterosexual partners of HIV-positive individuals, suggest that increased NK cell activity may be associated with resistance to HIV infection [28]–[32]. Epidemiological and genetic studies have also shown that expression of immunoglobulin like receptor KIR3DS1 on NK cells, and its ligand HLA-Bw4-80I, are associated with slower disease progression [33], [34]. Furthermore, NK cells expressing KIR3DS1 can strongly inhibit HIV-1 replication in vitro [34]. However, contradictory reports show that NK cell activity may have no effect on controlling virus replication [35]. Recently, another report compared the relative importance of NK cells, CD8^+^T cells, B cells and target cell limitation in controlling acute SIV infection in rhesus macaques and suggested that NK cells have little impact on the death rate of infected CD4^+^ cells and that their net impact may increase viral load [36]. However, these studies typically use samples collected from peripheral blood or lymph nodes and overlook the roles of mucosae-resident NK cells in limiting HIV replication during the initial stages of infection. In this study, we used the rhesus macaque model to evaluate the quantitative and qualitative effects of acute and chronic SIV infection on mucosae resident NKp44^+^ILCs and NK cells.

## Materials and Methods

### Ethics statement

All animals were housed at the New England Primate Research Center of Harvard Medical School in accordance with the rules and regulations of the Committee on the Care and Use of Laboratory Animal Resources. Animals were fed standard monkey chow diet supplemented daily with fruit and vegetables and water ad libitum. Social enrichment was delivered and overseen by veterinary staff and overall animal health was monitored daily. Animals showing significant signs of weight loss, disease or distress were evaluated clinically and then provided dietary supplementation, analgesics and/or therapeutics as necessary. Animals were humanely euthanized using an overdose of barbiturates according to the guidelines of the American Veterinary Medical Association. All studies reported here were performed under IACUC protocol #04637 which was reviewed and approved by the Harvard University IACUC.

### Animals and SIV infections

**Figure 1:**
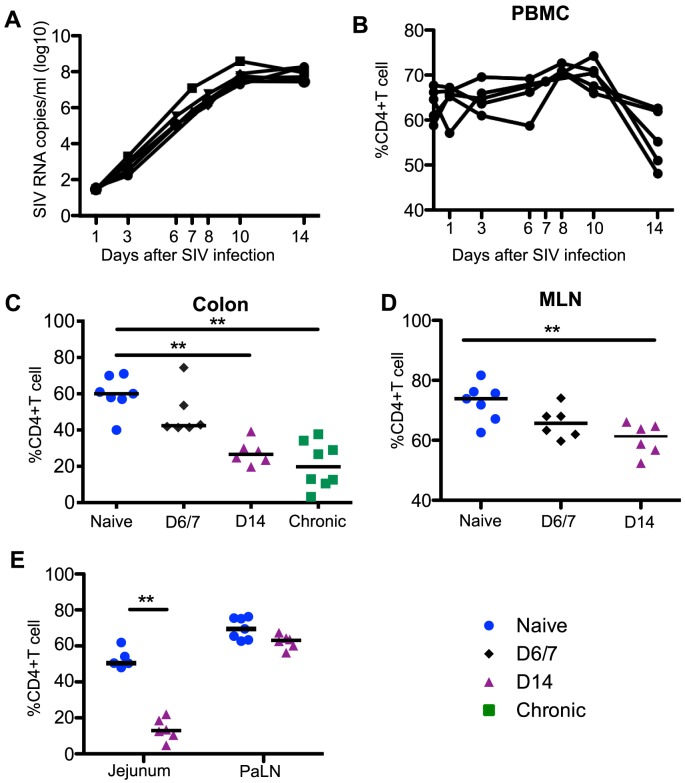
Plasma viremia and CD4^+^T cell dynamics in SIV infection. (A) Kinetics of plasma viral load during acute SIV infection. Percentages of CD4^+^T cells among total CD3^+^T cells in blood (B), colon (C), MLN (D), and jejunum and PaLN (E). Statistical comparisons are between naïve and acutely infected macaques at the indicated time points. Mann-Whitney *U* test; * *p*<0.05, ** *p*<0.01, *** *p*<0.001. MLN and PaLN; mesenteric and pararectal/paracolonic lymph nodes, respectively. Samples are from naïve macaques (n = 7) and those sacrificed at day 6/7 (n = 6), day 14 post-infection (n = 6), and those sacrificed in chronic disease (n = 8).

A total of twenty-six Indian rhesus macaques were analyzed in this study, including six SIV-naïve, twelve acutely and eight chronically infected with SIVmac239. For some tissues (i.e., blood, colorectal biopsy tissue), pre-infection data are grouped with naïve samples for cross-sectional comparisons. Most animals were infected intravenously except for 6 animals sacrificed at days 6 or 7, which were infected intravaginally. Infection was verified by plasma virus quantification by RT-PCR (see Fig. 1A) or by immunohistochemistry to SIV antigens (used in day-6/7 infection sacrifices, to be published elsewhere). Chronically SIV-infected macaques were infected between 162 and 707 days, with a median duration of 308 days. Chronic viral loads at time of necropsy were between 4.3 and 6.2 log_10_ copies of viral RNA/ml, plasma, with a median of 5.1 log_10_ copies of viral RNA/ml, plasma. CD4+ T cell frequencies in blood were between 54% and 22% of T cells, with a median of 39%. All animals were free of simian retrovirus type D and simian T-lymphotropic virus type 1, and were housed at the New England Primate Research Center or at the National Cancer Institute, National Institutes of Health. All animals were housed and cared for in accordance with the American Association for Accreditation of Laboratory Animal Care standards. All animal procedures were performed according to protocols approved by the Institutional Animal Care and Use Committee of Harvard Medical School and the National Institute of Allergy and Infectious Diseases, National Institutes of Health.

### Tissue collection and processing

Macaques were humanely euthanized at indicated time points and tissues were collected from colon, jejunum, mesenteric lymph node (MLN) and pararectal/paracolonic lymph node (PaLN). In a sub-group of the acutely infected animals, pre-infection (day -14) lymph node and colorectal biopsies were taken. Mucosal tissues were collected and lymphocytes isolated by mechanical and enzymatic disruption as described previously [23], [37], [38]. Total peripheral blood mononuclear cells were isolated from EDTA-treated venous blood by density gradient centrifugation over lymphocyte separation media (MP Biomedicals, Solon, OH) and a hypotonic ammonium chloride solution was used to lyse contaminating red blood cells.

### Antibodies and flow cytometry

Antibodies to the following antigens were included in this study and except where noted, all were obtained from BD Biosciences: α4β7-APC (clone A4B7, NHP reagent resource), active-caspase-3-Alexa647 (clone C92-605), CCR7-Alexa700 (clone 150503, R&D Systems), CD3-APC-Cy7 (clone SP34.2), CD4-FITC (clone L-200), CD16-Alexa-700 (clone 3G8), CD45-FITC (clone D058-1283), CD45-PerCp-Cy5.5 (clone Tu116), CD56-PE-Cy7 (clone NCAM16.2), CD62L-FITC (clone SK11), CXCR3-PE-Cy5 (clone 1C6), HLA-DR-PE-Texas Red (clone Immu-357, Beckman-Coulter), NKG2A-PE (clone Z199, Beckman-Coulter), NKG2A-Pacific Blue (clone Z199, in-house custom conjugate, Beckman-Coulter), NKp44-PerCp-Cy5.5 (clone Z231, Beckman-Coulter), Ki67-FITC (clone B56), Perforin-Pacific Blue (in-house custom conjugate, clone Pf-344, Mabtech). Flow cytometry acquisitions were performed on an LSR II (BD Biosciences, La Jolla, CA) and FlowJo software (version 9.6.4, Tree Star Inc., Ashland, OR) was used for all analyses. Pestle (Version 1.6.2) and SPICE (Version 5.1) were used for multi-parametric analyses.

### TruCount assay

TruCount flow cytometric assays for absolute CD4+ T cell counts were performed as previously described [39].

### Luminex

Cytokine concentrations in plasma were determined in a custom luminex assay as previously described [40]. Quantification of cytokines in mucosal washes was performed using a modified assay Millipore 23-plex non-human primate luminex kit platform. Briefly, colon or jejunum tissues collected at necropsy were diced into 3 mm pieces in R10 collection media. Aliquots of the cell-free media and plasma were snap-frozen for subsequent assays. Plates were read on a Bio-Rad 200 Bio-Plex system according to the manufacturer's suggested protocol. One hundred beads per regions were collected and results were optimized according to Bio-Rad software presets.

### NK-stimulation assay

Mononuclear cells were stimulated with phorbol myristate acetate (PMA, 50 ng/mL) and ionomycin (1 ug/mL) or cultured in medium (RPMI 1640 containing 10% FBS) alone. Anti–CD107a (PerCp-Cy5, clone H4A3) was added directly to each of the tubes at a concentration of 20 µl/ml, and Golgiplug (brefeldin A) and Golgistop (monensin) were added at final concentrations of 6 µg/ml. After culture for 12 hours at 37°C in 5% CO_2_, cells were surface stained then permeabilized (Caltag Fix & Perm) and stained intracellularly with anti-IL-17 (APC conjugate, clone eBio64DEC17, eBioscience), anti-IFN-γ (PE-Cy7 conjugate, clone B27; Invitrogen), and anti-TNF-α (Alexa700 conjugate, clone Mab11).

### In vitro ILC assay

NKp44+ ILCs from MLN of SIV-naïve rhesus macaques were stimulated overnight with either rhesus macaque IL-12 (50 ng/ml), human IL-1β (50 ng/ml), human IL-2 (1000 IU/ml) human IL-15 (50 ng/ml), or human IL-23 (50 ng/ml) (all from R&D Systems). After culture, cells were analyzed for intracellular expression of caspase-3 or RORγt (clone AFKJS-9, eBioscience).

### Plasma virus load quantification

Plasma SIV RNA copy numbers were determined using a standard quantitative real-time RT-PCR assay based on amplification of conserved gag sequences as described previously [41]. Tissue vDNA and vRNA quantifications were performed only on animals sacrificed at day-6/7 post-infection as described previously [42]. Briefly, 1-3 separate sections from duodenum, jejunum, ileum, cecum, colon, and rectum were examined and only 1 of 6 animals had detectable virus. Infection was confirmed by immunohistochemistry to SIV antigens in spleen or mucosal tissues (to be published elsewhere), but the undetectable vDNA and vRNA suggest infection in the GI tract was likely still focal at this early time point.

### Statistical analyses

All statistical and graphic analyses were performed using GraphPad Prism 6.0 software (GraphPad Software Inc., La Jolla, CA). Nonparametric Mann-Whitney *U* tests were used where indicated, and a *P* value of <0.05 (by a 2-tailed test) was considered statistically significant.

## Results

### Rapid and massive depletion of NKp44^+^ILCs in the intestinal mucosae during acute SIV infection

Acute lentivirus infections are characterized by high levels of viremia coupled to a dramatic loss of CD4^+^ T cells in the GI tract [3], [4]. In our cohort of acutely SIV-infected macaques, plasma viremia peaked between days 10 to 14 (Fig. 1A), and mucosal CD4^+^ T cells were significantly depleted by day 14 in jejunum, colon, and MLN tissues when compared to naïve controls and remained reduced in chronic infection (Fig. 1C-1E). The frequencies and absolute numbers of peripheral CD4^+^ T cell numbers were also significantly reduced by day 14 post-infection (Fig. 1B, S1 Figure).

**Figure 2:**
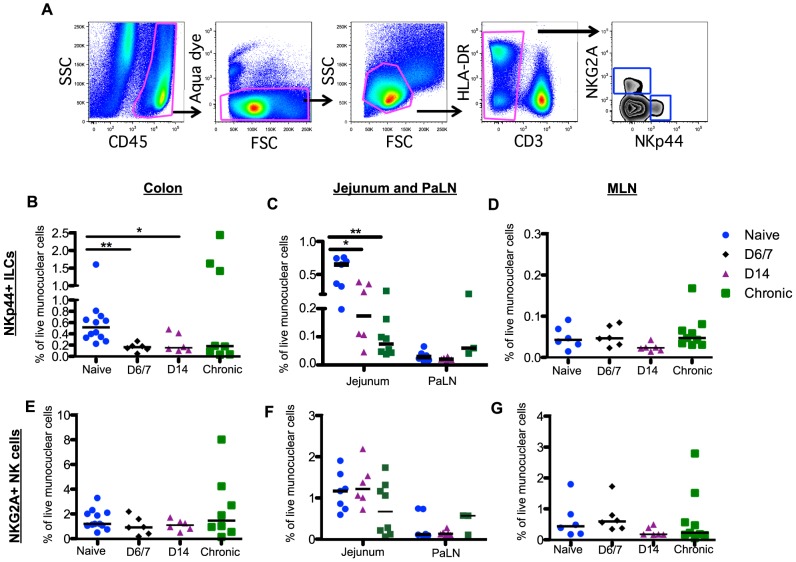
Rapid and massive depletion of NKp44^+^I LCs in intestinal mucosae after SIV infection. (A) Representative gating strategy to identify NKp44^+^ILCs and NKG2A^+^ NK cells. After gating on CD45^+^ leukocytes to exclude contaminating epithelial cells, dead cells and debris were excluded by using a vital stain. Among live CD45^+^CD3^–^ mononuclear cells, NKp44^+^ILCs and NK cells were identified by mutually exclusive expression of NKp44 and NKG2A, respectively. Frequencies of NKp44^+^ILCs among mononuclear cells in colon (B), jejunum and PaLN (C), and MLN (D) were compared between naïve, acute and chronically SIV-infected macaques at the indicated time points. Frequencies of NKG2A^+^NK cells among mononuclear cells in colon (E), jejunum and PaLN (F), and MLN (G) were compared between naïve, acute and chronically SIV-infected macaques at the indicated time points. Samples are from naïve macaques (n = 12) and those sacrificed at day 6/7 (n = 6), day 14 post-infection (n = 6), and those sacrificed in chronic disease (n = 8). Data from pre-infection colorectal biopsies are also grouped with naïve animal data for cross-sectional analyses.

Unlike T cells, innate immune responses in the GI tract during acute SIV infection are poorly understood. We have previously reported that chronic SIV infection resulted in depletion of NKp44^+^ILCs and classic NKG2A^+^NK cells in GI tract large bowel tissues [23]. However, little is currently known about the effects of SIV infection on ILCs and NK cells in other sites of the GI tract, and nothing is known about the effects of acute infection. In the present planned-euthanasia study, we found, as early as one week after infection, there was up to a 3-fold decrease of NKp44^+^ILCs (Fig. 2A) in colons from acutely infected compared to naïve macaques (Fig. 2B). Using pre-infection colorectal biopsy samples, we also analyzed changes in NKp44^+^ILCs in individual animals sacrificed at day 14 post-infection and found that all 6 macaques exhibited NKp44^+^ILC depletion in colorectal tissue (S2A Figure). Furthermore, NKp44^+^ILCs were profoundly and persistently lost from jejunum in both acute and chronic SIV infections with 4- and 9-fold decreases, respectively (Fig. 2C). Frequencies of NKp44^+^ILCs in MLN and PaLN in either acute or chronic infection were unchanged compared to naïve macaques (Fig. 2C, 2D), suggesting loss of NKp44^+^ILCs may be compartmentalized. Furthermore, loss of ILCs did not correlate with viral load.

Although the percentage of NK cells in circulation was significantly decreased by day 14 post-infection (S2C Figure), in stark comparison to the massive loss of mucosal NKp44^+^ILCs there were no significant changes in the frequency of NK cells throughout the GI tract (Fig. 2E–2G). Furthermore, no change in NK cell numbers in colorectal tissues from individual animals pre- and post-infection was observed (S2B Figure). There was also no change in the distribution of CD56^+^CD16^-^ (CD56^+^); CD56^-^CD16^+^(CD16^+^); and CD56^+^CD16^+^, and CD56^-^CD16^-^ NK cell subsets during acute infection (S3 Figure).

### SIV induces profound apoptosis of NKp44^+^ILCs in intestinal mucosae

**Figure 3:**
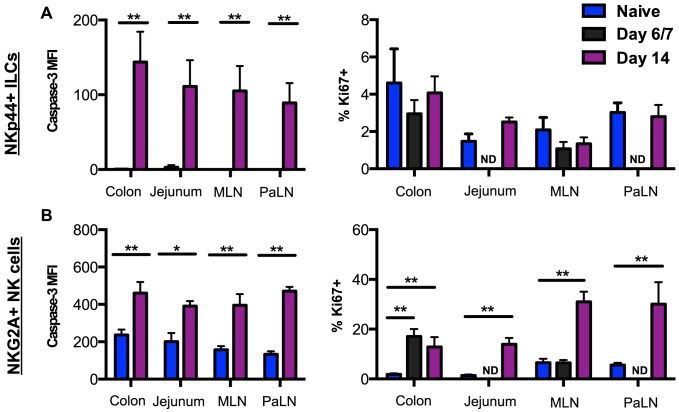
SIV-induced turnover of NK cells and ILCs in intestinal mucosae. (A) Median fluorescence intensity (MFI) of caspase-3 expression and frequency of Ki67 expression in NKp44^+^ILCs from mucosal tissues were compared between naïve and acutely SIV-infected macaques. (B) Expression of caspase-3 and Ki67 in NK cells from mucosal tissues were compared between naïve and acutely SIV-infected macaques. Samples are from naïve macaques (n = 6) and those sacrificed at days 6/7 (n = 6) or 14 post-infection (n = ). Mann-Whitney *U* test; * *p*<0.05, ** *p*<0.01. ND, not done.

To begin to address the mechanism of depletion of NKp44^+^ILCs in intestinal mucosae during acute SIV infection, we analyzed the expression of the active form of the apoptotic molecule casapase-3 and the proliferation marker Ki67. As shown in Fig. 3A, NKp44^+^ILCs from GI tissues had little to no expression of active-caspase-3 in naïve macaques, but increased greater than 100-fold in macaques sacrificed by day 14 post-infection. Interestingly, NKp44^+^ ILCs had no detectable change in Ki67 expression. By comparison NK cells had significant increases in both active-caspase-3 and Ki67 expression following infection (Fig. 3B). We have previously reported similar findings for ILCs in chronic SIV infection [23], but no correlation was found between caspase-3 levels and ILC frequencies. These disparate changes in turnover could account for the massive loss of NKp44^+^ILCs while NK cells were maintained in tissues.

### Lack of altered trafficking repertoires of NK cells during acute SIV infection

Although NKp44^+^ILCs are mucosae-resident, trafficking in and out of the mucosae could influence the numbers of NKG2A^+^ NK cells. We next investigated whether turnover of NK cells in tissues after infection could be affected by trafficking to and within the GI tract. We have previously demonstrated that chronic SIV infection induces NK cell trafficking to the gut mucosae, characteristically by significant up-regulation of the gut-homing marker α4β7 on peripheral NK cells with concomitant down-regulation of the lymph node-homing markers, CCR7 and CD62L [23], [43]. Here, we compared the expression of each of these trafficking markers on NK cells in blood and mucosal tissues from naïve, acute and chronically SIV-infected macaques. As shown in S4 Figure, compared with uninfected macaques NK cells in both the circulation and tissues had no statistically significant differences in α4β7 expression except for PaLN. Similarly, no significant change in expression of CCR7, CD62L, or CXCR3 was detectable on NK cells from any GI tissue during acute infection. However, during chronic infection we observed a dramatic down-regulation of CXCR3 in colon, jejunum, and MLN. These data combined with previous observations from our laboratory and others [23]–[25] suggest that while SIV infection may alter NK cell trafficking to the mucosae, it likely occurs in chronic rather than acute infection (S4 Figure).

### Increased cytotoxic potential and multifunctional cytokine production by NKp44^+^ILCs in the GI tract during SIV infection

**Figure 4:**
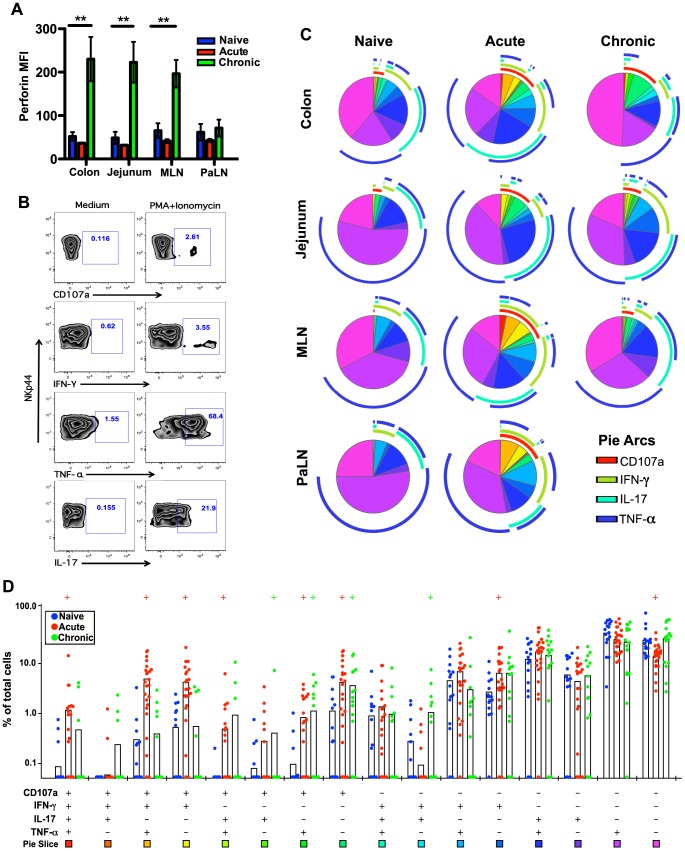
Comparison of NKp44^+^ILC functions within naïve, acute and chronically SIV-infected macaques. (A) Median fluorescence intensities (MFI) of perforin expression in NKp44^+^ILCs ex vivo. (B) Representative flow cytometry plots demonstrating cytokine-secretion profiles of mucosal NKp44^+^ILCs following mitogen stimulation. (C) Multiparametric analyses on the data shown in (B) were performed with SPICE 5.0 software. Pies indicate means of 6 to 8 animals per group for each multifunction and arcs show overlap of individual functions. (D) Combined tissue bar comparisons of individual multifunctions. Multifunctions are indicated by rainbow color boxes beneath each combination and correspond to the same colored pie slices in (C). Statistically significant differences between naïve versus acute (red) or naïve versus chronic (green) are indicated; Student's *t* test. Samples are from naïve macaques (n = 6), those sacrificed at day 14 post-infection (n = ), and animals sacrificed in chronic disease (n = 8).

Our data show that acute SIV infection induces a massive depletion of NKp44^+^ILCs from the intestinal mucosae. We next asked whether there is any impact on the functionality of ILCs or NK cells during acute infection. We first analyzed a surrogate marker of cytotoxic potential, intracellular expression of the cytolytic granule, perforin. Our previous research suggested that while NKp44^+^ILCs are generally noncytolytic, under inflammatory conditions they can acquire killing activity [23]. As shown in Fig. 4A, mucosal NKp44^+^ILCs from acutely SIV-infected macaques had expression of intracellular perforin at low levels not significantly different from naïve animals. However, during chronic SIV infection intracellular perforin increased 4-fold in NKp44^+^ILCs within colon, jejunum, and MLN.

We then investigated cytokine production and degranulation by NKp44^+^ILCs as true measures of functionality. As shown in Fig. 4B–D, following mitogen stimulation, NKp44^+^ILCs in colon, MLN, and PaLN from acutely SIV-infected macaques had more than 3-fold increases in expression of CD107a^+^ and IFN-γ compared to naïve animals. Furthermore, multiparametric analysis revealed NKp44^+^ILCs in the GI tract from either acute or chronic SIV infected macaques have increased multifunctional capacity – upregulating CD107a and producing increased IFN-γ and TNF-α compared with naïve macaques (Fig. 4C, 4D). Interestingly, NKp44+ ILCs maintained their ability to produce IL-17 in acute, but not chronic SIV infection (S5A Figure). Furthermore, the acute loss of ILCs appeared to be associated with an acute loss of IL-17 in plasma and overall reduction in systemic and mucosal IL-17 in chronic disease (S5B Figure and S5C Figure), well in line with previous observations [23], [25], [26].

### Increased cytotoxic potential of NK cells in the gastrointestinal tract during SIV infection

Thus far, it is still unclear what role NK cells play in controlling SIV replication in GI tract. NK cells can directly inhibit virus infectivity and lyse infected cells by releasing perforin and granzyme. Here, we investigated the expression of intracellular perforin in mucosal NK cells *ex vivo*. We found that mucosal NK cells in all tissues significantly upregulated perforin expression by two weeks post-infection (S6 Figure). Furthermore, perforin expression remained upregulated in chronic infection, suggesting heightened cytolytic potential throughout the disease course. Interestingly, perforin expression was increased in all subpopulations of NK cells except CD16^+^NK cells, which already had the highest levels of expression (S4 Figure). However, the level of perforin expression in mucosae resident NK cell did not have a statistically significant relationship with plasma viral load.

**Figure 5:**
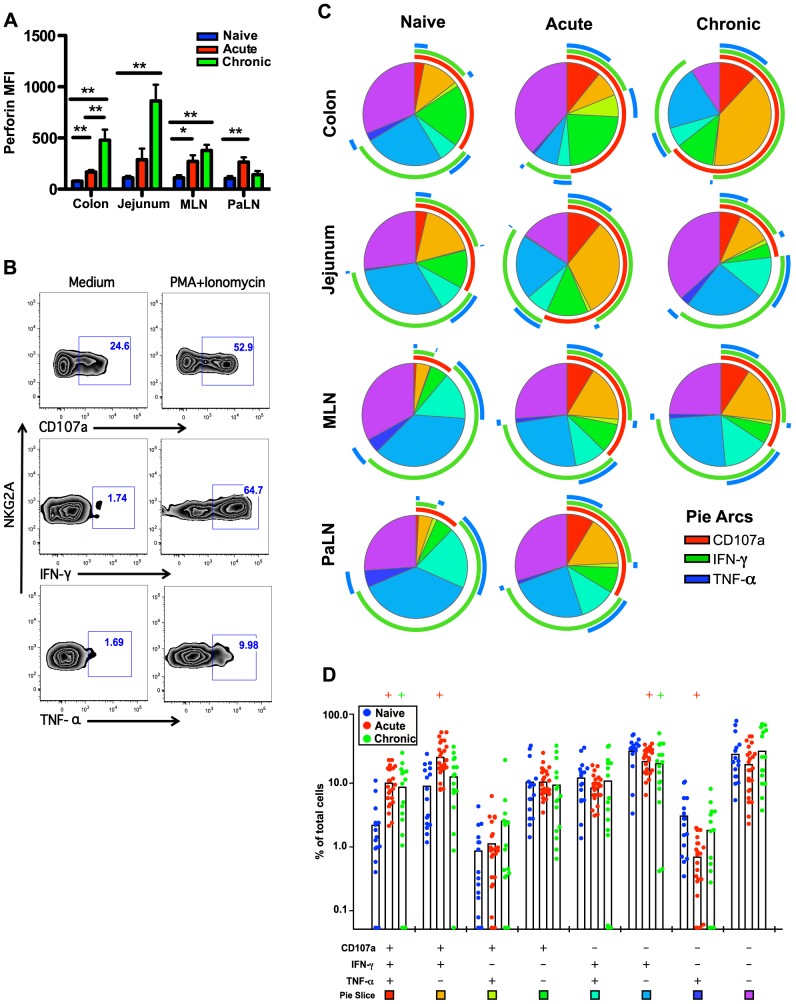
Comparison of NKG2A^+^NK cell functions within naïve, acute and chronically SIV-infected macaques. (A) Median fluorescence intensities (MFI) of perforin expression in NKG2A^+^NK cells *ex vivo*. (B) Representative flow cytometry plots demonstrating cytokine-secretion profiles of mucosal NKG2A^+^ILCs following mitogen stimulation. (C) Multiparametric analyses on the data shown in (B) were performed with SPICE 5.0 software. Pies indicate means of 6 to 8 animals per group for each multifunction and arcs show overlap of individual functions. (D) Combined tissue bar comparisons of individual multifunctions. Multifunctions are indicated by rainbow color boxes beneath each combination and correspond to the same colored pie slices in (C). Statistically significant differences between naïve versus acute (red) or naïve versus chronic (green) are indicated; Student's *t* test. Samples are from naïve macaques (n = 6), those sacrificed at day 14 post-infection (n = 6), and animals sacrificed in chronic disease (n = 8).

Lastly, we tested the functionality of mucosae-resident NK cells. As shown in Fig. 5B, following stimulation with PMA/ionomycin acutely SIV-infected macaques had 2-fold increases in CD107a and 1.5-fold higher IFN-γ secreting NK cell in colon. Multi-parametric analysis showed these mucosae resident NK cells from both acute and chronic SIV-infected macaques have increased percentages of multifunctional cells which were positive for CD107a, IFN-γ, and TNF-α, compared with naïve macaques (Fig. 5C, 5D).

### Increased inflammatory mediators in acute SIV infection associated with ILC modulation

**Figure 6:**
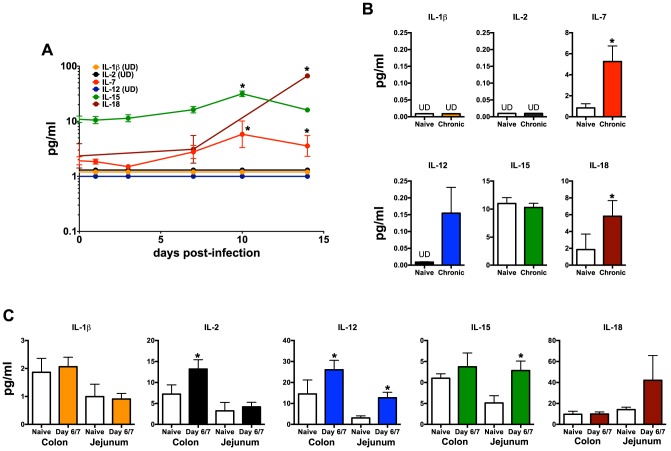
Quantification of inflammatory cytokines in SIV-infected macaques. (A) Longitudinal cytokine levels in plasma during acute SIV infection. (B) Comparison of plasma cytokine levels in naïve and chronically SIV-infected macaques. (C) Cytokine concentrations in gastrointestinal washes from naïve and SIV-infected macaques sacrificed at days 6/7 post-infection as described in the Methods. Statistically significant differences between pre-infection and post-infection samples (A) were determined by Student's *t* test. Mann-Whitney *U* test was used for cross-sectional comparisons (A & B). Only statistically significant *p* values are shown; *, *p*<0.05. Samples are from naïve macaques (n = 6), those sacrificed at day 6/7 post-infection (n = 6), and animals sacrificed in chronic disease (n = 8). UD, undetectable.

**Figure 7:**
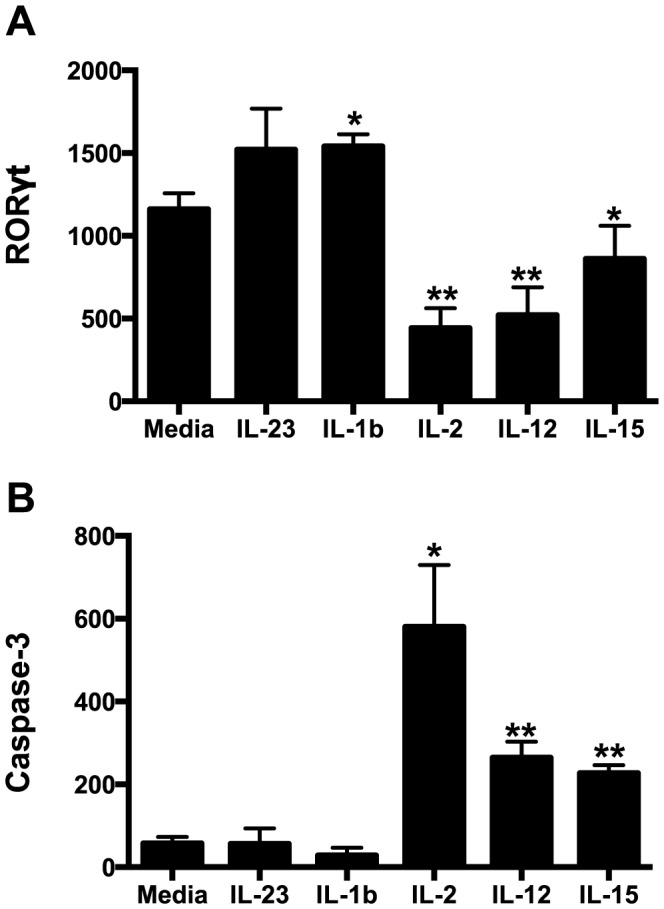
Intracellular RORγt and caspase-3 levels in cytokine cultured NKp44+ ILCs. NKp44+ ILCs were cultured overnight in various cytokines and RORγt (A) and caspase-3 (B) were quantified intracellularly by flow cytometry. Bars represent mean ± SEM of 5 independent experiments. Student's *t* test was used to compare media control to each cytokine-treated culture; * *p*<0.05, ** *p*<0.01. MFI, median fluorescence intensity.

Our data demonstrate that not only are NKp44+ ILCs depleted in SIV infection, but they also have altered phenotypes and functions. NKp44+ ILCs in macaques are most likely analogous to ILC3, but in infection their functional repertoire resembles that of ILC1. Moreover, ILCs stem from a common precursor are thought to exhibit varying degrees of plasticity, whereby environmental cues can convert ILC3 to ILC1 and vice versa. We next evaluated whether changes in the inflammatory environment due to infection might favor ILC1 development and thus explain the loss of ILC3. Interestingly we found that IL-7, which is necessary for both ILC1 and ILC3 development was elevated in acute infection and remained so during chronic disease (Fig. 6). IL- β which favors ILC3 development was either undetectable or remained unchanged. In contrast, IL-2, IL-12, IL-15, all of which favor ILC1 development, were all elevated at various points in disease. Indeed, in *in vitro* experiments culturing NKp44+ ILCs with various cytokines, IL-23 and IL- β both promoted RORγt expression, IL-2, IL-12, IL-15 suppressed expression and increased apoptosis (Fig. 7A, 7B). Overall these data suggest that the inflammatory environment found in both acute and chronic SIV infection depletes the ILC3 population by simultaneously inducing apoptosis and favoring development of ILC1.

## Discussion

In this study, we sought to explore the impact of acute SIV infection on ILCs and NK cells in the GI tract and draining lymph nodes. We observed a rapid and massive depletion of NKp44^+^ILCs, but not NK cells, as early as one week following SIV infection, at least partially attributable to increased apoptosis. Furthermore, we found both NKp44^+^ILCs and mucosae-resident NK cells had altered functional repertoires characteristic of heighted cytotoxicity. To the best of our knowledge, these data are the first to demonstrate a SIV-induced rapid and permanent loss of NKp44+ ILCs in the gut. The full implications of this novel aspect of lentiviral pathogenesis remain unclear.

Previously, based on a limited study focusing on colorectal tissue only, we reported that NKp44^+^ILCs were depleted during chronic SIV infection [23]. Soon after other groups corroborated our findings by reporting that that IL-17-secreting ILCs were lost from jejunum and in SIV-infected rhesus macaques [24], [25]. However, prior to the current study it was unknown kinetically when NKp44^+^ILCs were depleted and whether this phenomenon occurred in other mucosal tissues. In this new study we report that NKp44^+^ILCs were massive depleted from GI tissues as early as the first week after SIV infection, and remained suppressed during chronic disease. We also found that NKp44^+^ILCs had dramatically increased levels of apoptosis, without any change in proliferation rate, a plausible explanation for the net loss of cells. Our in vitro analyses clarified that apoptosis was due, at least in part, to increased inflammatory cytokines IL-2, IL-12, and IL-15 in the gut (Fig. 7). Furthermore, these new data demonstrate that the loss of NKp44+ ILCs is compartmentalized, since we observed no depletion in pararectal/paracolonic- and mesenteric-draining lymph nodes. Because we have demonstrated that NKp44^+^ ILC loss in the colorectum is partially due to increased inflammation (Fig. 3, Fig. 6, & Fig. 7) [23], [44], it will be of interest in future studies to determine if these mediators are not increased in lymph nodes. While a specific mechanism is unclear, ILC loss is unlikely due to infection as previous studies from our laboratory have shown [23].

NKp44^+^ILCs are most likely analogous to RORγt^+^ ILC3 [23] that play a significant role in mucosal homeostasis and intestinal integrity. The rapid and significant depletion of NKp44^+^ILCs after acute SIV infection suggests ILC loss might be directly or indirectly related to the breakdown of the gut epithelium, a hallmark of HIV/SIV disease [45], [46]. Indeed, a recent study in mice suggests ILC3 promote anatomical containment of lymphoid-resident bacteria through induction of antimicrobial peptides, and depletion of ILCs results in peripheral dissemination of commensal bacteria and systemic inflammation [11]. Thus, loss of ILCs might also have a direct role in gut breakdown and subsequent microbial translocation. Indeed, Klatt and colleagues [25] have previously shown that loss of IL-17/22 production by ILCs in SIV-infected macaques is associated with loss of epithelial integrity in the gut. ILC3 also exhibit significant regulation of myeloid and T regulatory cells in the gut through production of GM-CSF, and gastrointestinal dysfunction in SIV infection could be partially attributed to this mechanism [47]. NKp44^+^ILCs in the GI tract had further alterations shifting to multifunctional cells, including production of IFN-γ and TNF-α and gaining cytotoxic potential. Some human and mouse studies have actually shown IL-17- and IL-22- secreting ILC3 can become IFN-γ-secreting cells with T-bet acquisition induced by bacterial infection [48]–[50]. Our data demonstrating suppression of RORγt related to increased inflammatory mediators in the gut could explain this overall change in ILC phenotype and functional conversion of NKp44^+^ILCs to a proinflammatory and/or hypercytotoxic repertoire could exacerbate the pathogenesis of SIV infection.

Increased NK cell activity has been reported during HIV infections [51], [52] and we demonstrate a similar effect in both acute and chronic SIV infections. However, it has been difficult to ascribe a specific role for NK cells in either control of virus replication, or, alternatively, pathogenesis. Partly due to the fact that current strategies for NK cell depletion are incomplete [53], [54]. NK cells are known to lyse both HIV- and SIV-infected cells, but to what extent this occurs in vivo is unclear [34], [51], [52], [55]–[59]. Thus far, most studies have also focused on circulating NK cells, but in this study we investigated mucosae-resident NK cell responses during early SIV infection. The broad activation of NK cells in acute disease that persisted into chronic infection suggests NK cells are highly responsive to ongoing virus replication, a notion supported by their decreased activation during antiretroviral therapy [60]–[62]. Recently it was reported that NK cells from individuals carrying the KIR3DL1 receptor had greater tri-functional responses (TNF-α, CD107a and IFN-γ) [63], and linked these responses to greater control of viremia. Similarly, we found that during acute SIV infection, NK cells acquired a similar multifunctional phenotype. It will be of interest in future studies to determine if such a functional repertoire could be associated with greater control of focal virus replication in the gut or abortive infections.

In summary, we show acute SIV infection has significant, yet somewhat disparate, effects on two populations of mucosal innate lymphocytes. Although the precise niche of NKp44+ ILCs in primates is yet to be elucidated, it is tempting to speculate that the early and massive loss of these cells constitutively producing IL-17 and IL-22 could have a significant impact on mucosal homeostasis. Furthermore, the increases in cytotoxicity and inflammatory cytokine production by both ILCs and NK cells during acute infection are likely to contribute to the massive apoptosis and dysregulation in the gut. Regardless, given the profound impact of acute SIV infection on both cell types, further study into both the underlying mechanisms and clinical consequences are warranted.

## Supporting Information

S1 Figure **Absolute counts of circulating CD4^+^ T cells during acute SIV infection.**
(EPS)

S2 Figure **Dynamics of NKp44^+^ILCs and NK cells in individual animals during acute SIV infection.** (A) Frequencies of NKp44^+^ILCs among mononuclear cells in colorectal tissues were compared between pre-infection biopsies and at day 14 post-infection sacrifice. (B) Frequencies of NK cells among mononuclear cells in colorectal tissues were compared between pre-infection biopsies and at day 14 post-infection sacrifice. (C) Means and standard deviations of circulating NK cell frequencies from six animals following acute SIV infection. Wilcoxon matched pairs test; * *p*<0.05.
(EPS)

S3 Figure **Frequencies of NK cell subsets among total NK cells in mucosal tissues from naïve and acutely SIV-infected macaques.** Based on CD16 and CD56 expression, four subpopulations are identified among the total NK cell, CD56^+^CD16^-^ (CD56^+^); CD56^-^CD16^+^(CD16^+^); and CD56^+^CD16^+^ (double positive [DP]), and CD56^-^CD16^-^ (double negative [DN]). No changes were observed among tissues following infection.
(EPS)

S4 Figure **Expression of trafficking markers on circulating NK cells.** Median fluorescence intensities (MFI) of α4β7 (A), CD62L (B), CCR7 (C), and CXCR3 (D) expression on NK cells in circulating and tissues were compared between naïve and acute SIV-infected macaques.
(EPS)

S5 Figure **IL-17 production in SIV infection.** (A) Monofunctional production of IL-17 by NKp44+ ILCs as shown in Fig. 4. IL-17 concentrations in mucosal washes (B) and plasma (C) during SIV infection. UD, undetectable in all animals.
(EPS)

S6 Figure **MFI of perforin expression in NK cell subsets in tissues.**
(EPS)
